# A New Explanation for the Frog-in-the-Pan Phenomenon Based on the Cognitive-Evolutionary Model of Surprise

**DOI:** 10.3390/bs13010007

**Published:** 2022-12-22

**Authors:** Dapeng Liang, Mengting Liu, Yang Fu, Jiayin Sun, Hongyan Wang

**Affiliations:** 1 School of Management, Harbin Institute of Technology, Harbin 150001, China; 2 School of Humanities and Social Sciences, Harbin Institute of Technology, Harbin 150001, China; 3 School of Mathematical Science, Heilongjiang University, Harbin 150080, China

**Keywords:** frog-in-the-pan, surprise, cognitive-evolutionary model of surprise, reinforcement learning model, decision making

## Abstract

The frog-in-the-pan (FIP) phenomenon suggests that investors are more sensitive to abrupt price changes than gradual price changes in the stock market. Based on the cognitive-evolutionary model of surprise and the reinforcement learning model, this paper provides a new explanation for the FIP phenomenon in that this phenomenon could be explained by the elicitation of surprise emotion. We predict that when a change substantially and abruptly occurs, the significant prediction error triggers participants’ surprise, which makes participants more sensitive to the change. To ascertain these hypotheses, we recruited 109 participants and compared participants’ learning rates and surprise responses under different contexts. We observed that participants’ learning rate soared when the prediction error was large enough to trigger surprise emotion under abruptly changed conditions and confirmed that the FIP phenomenon could be explained by the elicitation of surprise emotion. In a word, this research demonstrates the significant role of surprise emotion in the decision-making process.

## 1. Introduction

The efficient market hypothesis (EMH) [1] presumes that investors are rational [2,3,4] and use all available information to form “rational expectations” about the value of assets [5]. However, more and more studies have confirmed that investors are irrational [6,7,8,9,10,11] and exhibit information bias [12]. For example, investors react differently to stock price changes. And they tend to be less sensitive to gradual changes than dramatic changes [13]. Inspired by the boiling-frog anecdote, Da et al. [14] found that investors were insensitive to information arriving continuously in small amounts while more sensitive to information arriving at discrete time points in large amounts (frog-in-the-pan phenomenon, FIP). A lack of attention could explain this phenomenon. However, some research suggested that this explanation remains open to question [15].

The present research sought to provide a new explanation for the FIP based on the cognitive-evolutionary model of surprise [16]. The cognitive-evolutionary model of surprise proposes that surprise emotion is activated when the new information disconfirms the belief. When people feel surprised, they tend to update their beliefs and put more weight on the new information. In other words, people are more likely to rely on the newly received information during estimation when they are surprised. Based on this theory, we propose that under gradually changing conditions, the difference between the participants’ beliefs and the new information (i.e., the prediction error) is relatively small, which fails to trigger surprise emotion. Therefore, participants are likely to be insensitive to the new information. However, when changes are abruptly introduced, the prediction error is large enough to elicit surprise emotion and induce belief updating. Thus, participants tend to be more sensitive to the new information. We conducted a brand-new estimation task adapted from Nassar and his colleagues’ [17] predictive-inference task to ascertain this explanation. This task included two main conditions: the gradually changed condition and the abruptly changed condition. First, we used the learning rate from the reinforcement learning model (RL model) to quantify the effect of new information on participants’ estimation under the gradually changed condition and the abruptly changed condition. We found that participants tended to rely more on new information to update their beliefs in the latter case. Second, we conducted a segmented regression to verify that the learning rate would soar when the prediction error is large enough to elicit surprise emotion. And we observed that participants tended to make a significant prediction error under the abruptly changed condition. Lastly, we proved that surprise emotion was induced when changes were abruptly introduced by comparing RTs under the gradually changed condition and the abruptly changed condition.

This research makes several contributions. First, we provide a new explanation for the FIP phenomenon based on the cognitive-evolutionary model of surprise and the RL model. Second, we call attention to how surprise affects evaluation in the decision-making process, which is also the main contribution of the present study.

## 2. Literature Review

### 2.1. Previous Explanations for the FIP Phenomenon

Da and his colleagues were the first researchers to propose the FIP phenomenon in the financial field [18]. Before Da et al. research, however, some researchers had noticed that people were likely to be imperceptive to gradual adjustments while perceptive to abrupt adjustments in daily life.

Gino and Bazerman found that the FIP phenomenon existed in unethical behavior [13]. They set two roles in their research, the first one was estimators and the second one was approvers. Estimators were required to estimate the coins in a jar repeatedly. If an estimation was accepted by approvers, the estimator would receive 8% of the estimation. Therefore, deliberately giving high estimation was unethical behavior in this experiment. The result demonstrated that participants were more receptive to progressive unethical behavior and less receptive to abrupt unethical behavior, which indicated that participants showed greater sensitivity to sudden unethical behavior and greater insensitivity to gradual unethical behavior. The plausible explanation was that people tended to use previous behavior as the standard to evaluate new behavior. Therefore, if the previously observed behavior was acceptable, then a similar or slightly different behavior would be tolerated as well. In addition, unethical behavior might become routine, and people would be inattentive to it [13]. In a word, gradual unethical behavior was difficult to detect which led to such behavior being more easily accepted.

Based on Gino and his colleague’s research, Da et al. conjectured and confirmed that investors showed different sensitivity to different changes in magnitude [14]. Investors were more sensitive to sudden and substantial price changes than slow and progressive price changes. Da and his colleagues asserted that limited attentional resources could inhibit investors’ ability to process all available information immediately, which led investors to allocate more resources to dramatic changes and hence more sensitive to new changes [14]. To verify this conjecture, Da et al. conducted a Fama–MacBeth regression and observed that the discreteness of information was positively correlated with turnover as well as media coverage and press releases. In addition, Da et al. provided a two-period illustrative model with two types of investors. They demonstrated that rational investors processed all information immediately while FIP investors processed the information below the attention threshold with a delay. Therefore, a lack of attentional sources led to the FIP phenomenon.

Offerman et al. reported that the FIP phenomenon was also found in the public goods field [15]. Offerman et al. stated that the FIP phenomenon could be explained by the anchoring effect. When a person considered the situation approximately constant, he believed that the previous decision was optimal. When he detected a substantial change, he was motivated to change his decision. The “cost of thinking” may set a barrier for the person to frequently adjust his decision. Notably, Offerman and his colleagues proposed that the FIP phenomenon could not be explained by attention. With the public goods game as the background, Offerman and his colleagues designed two treatments: the single-task treatment and the dual-task treatment. In the single-task treatment, participants were only informed to finish a public goods game and pay attention to the change in the subsidy. While in the dual-task treatment, participants not only observed the adjustment in subsidy but also attended to another task. However, they found that participants responded similarly to the adjustments in subsidy in the single-task treatment as they did in the dual-task treatment. Therefore, the FIP phenomenon did not result from limited attention.

Yang also noticed that participants were more susceptible to a sudden and substantial change in an investment game [19]. She proposed that gradual changes were tentative while substantial changes were confirmative. As a result, participants responded to sudden changes quickly. Yang set two types of information: the first information included a sequence of minor changes while the second information included a substantial change. Although participants made similar investment decisions under different conditions, they took significantly less time to respond to the substantial changes. It suggested that participants were more susceptible to sudden and substantial adjustments.

In the research on extreme market events, Piccoli et al. asserted that investors were more likely to overreact to extreme events in the stock market [20]. Based on the research of Griffin and Tversky [21], Piccoli and his colleagues provided a plausible explanation for the FIP phenomenon. They stated that investors tended to pay attention to the extremeness of available evidence and pay less attention to the size of the evidence. As a result, investors were more likely to overreact to extreme events and underreact to less extreme but more frequent events.

In a word, the abovementioned research mainly explained the FIP phenomenon from cognitive bias, while few studies used surprise emotion as a plausible explanation for the FIP phenomenon.

### 2.2. Surprise Emotion

Surprise is one of six basic emotions [22]. The cognitive-evolutionary model of surprise proposes that we unconsciously, continuously, and automatically compare the current activated belief with the newly received information [16,23]. Surprise emotion will be elicited when we detect that the new information is significantly different from the existing belief [16,23,24,25,26,27,28,29]. The degree of surprise is determined by the discrepancy between the previous belief and the new information [23,30]. The cognitive-evolutionary model of surprise and a large number of studies have shown that surprise drives belief updating [16,17,31,32,33]. When surprise emotion is induced by the discrepancy between belief and new information, the feeling of surprise makes people come to realize that the previous belief is not supported by the new information [34]. As a result, people are likely to rely less on the original belief and more on new information to revise the belief.

Based on the cognitive-evolutionary model of surprise, we conjectured that people tend to be less sensitive to gradual changes because the discrepancy between people’s beliefs and the new information is small. As a result, people tend to be less sensitive to new information. However, when change is abruptly introduced, the discrepancy is large enough to induce surprise emotion, which drives people’s belief of updating. Therefore, people are likely to be more sensitive to new information. In brief, surprise emotion makes participants more sensitive to new information.

### 2.3. The Reinforcement Learning Model (the RL Model)

Even though the cognitive-evolutionary model of surprise demonstrates the role of surprise in learning and belief updating, it does not offer an algorithm to measure the effect of surprise on belief updating. Thus, we adopted the RL model [35] (a detailed description is shown in Section 4.4.1), which provides the quantitative frameworks for understanding adaptive behavior to demonstrate the role of surprise in the decision-making process.

In the RL model, the degree to which participants are affected by the new information is quantified by the learning rate [36,37]. McGuire et al. [33] and Nassar et al. [17], respectively, used the RL model to investigate participants’ learning behavior in a dynamic environment. They both confirmed that participants had a higher learning rate and showed a significant belief of updating when the stimulus was surprisingly adjusted.

## 3. Hypothesis

According to the RL model, a significant learning rate indicates that participants allocate more weight to the new information; a tiny learning rate suggests that the effect of new information on participants’ estimation is minimal. The FIP phenomenon indicates that participants are more likely to be insensitive to gradual changes and sensitive to abrupt changes. Based on the RL model, we conjectured that the learning rate was relatively low under the gradually changed condition, and the learning rate soared under the abruptly changed condition. Therefore, we proposed hypothesis 1 below.

Hypothesis 1: The learning rate is relatively low when participants face a sequence of gradual changes. However, the learning rate is high when participants encounter an abrupt change.

Hypothesis 1 indicates that participants are more sensitive to sudden adjustments and less sensitive to gradual adjustments in target value. Based on the cognitive-evolutionary model of surprise, we expected that the learning rate would soar when the prediction error is large enough to induce surprise emotion. In addition, participants tend to make a significant prediction error when the change abruptly occurs. As a result, participants accelerate learning the new information under the abruptly changed condition. To ascertain this reasoning, we proposed hypothesis 2 below.

Hypothesis 2: Participants’ learning rates are associated with their prediction errors. Under the abruptly changed condition, the learning rates show a significant value jump due to the large prediction error produced by the participants. In addition, the large prediction error is more likely to occur in abruptly changed trials.

In addition, previous works indicated RTs were elevated when people were surprised [16,25,27,38,39]. We tested this conclusion in this research. Specifically, we compared RTs in gradually and abruptly changed trials. By comparing RTs in these trials, we ascertained hypothesis 3 below.

Hypothesis 3: Participants are surprised by the significant prediction error in abruptly changed trials because RTs are elevated in these trials.

The remainder of this paper is organized as follows. Section 4 briefly describes our experimental design in this research. In Section 5, we present statistical evidence of our conjecture. Finally, in Section 6, we conclude and discuss our findings.

## 4. Materials and Methods

### 4.1. Experiment Overview

We developed a brand-new estimation task based on Nassar and his colleagues’ predictive-inference task to compare the learning rate and surprise response after a sudden and dramatic change and a series of gradual changes [17]. The experiment included two tasks. The first one was the estimation task and the second one was the reaction time (RT) task.

The estimation task aimed to compare participants’ sensitivity toward abrupt adjustments and progressive adjustments in target value. In this task, participants were required to predict the forthcoming numerical value (i.e., the target value, and its distribution is shown in Table 1) based on their previous beliefs. The target value was set to slightly fluctuate in gradually changed conditions and swing in abruptly changed conditions (shown in Table 1). We predicted that participants would be more susceptible to sudden changes. To verify our prediction, we recorded participants’ estimations and prediction errors (i.e., the gap between participants’ estimation and the target value) in each trial. By adopting the RL model, we calculated participants’ learning rates when the target value abruptly adjusted and progressively adjusted and compared the two learning rates to investigate their sensitivity toward abrupt adjustments and progressive adjustments.

The RT task was used to test whether participants were surprised by the large prediction error when the target value suddenly and dramatically varied. In this task, participants were informed to press the corresponding mouse button as quickly as possible. Participants not only observed a letter indicating whether they should press the left or right mouse button but also saw a green rectangle which represented the prediction error. The prediction error was the gap between the target value and their latest estimation, which would soar when the target value abruptly varied. We predicted that participants would be distracted by the large prediction error and spend more time pressing the corresponding button. As a result, RTs were extended when the target value suddenly and substantially adjusted. To test this prediction, we measured and compared participants’ reaction times after they saw the letter on the screen under different conditions.

### 4.2. Participants

One hundred and nine students participated in the experiment, including 56 undergraduates and 53 post-graduates. All gave written informed consent and received a monetary bonus for participation. Participants were paid a CNY 25 show-up fee and received up to CNY 20 monetary bonus depending on their performance. The internal Review Board of Management of Harbin Institute of Technology approved the experiment.

### 4.3. Material and Procedure

The experiment consisted of a practice block (20 trials) and two formal blocks (the gradually changed block/the abruptly changed block). The order of the two formal blocks was counterbalanced.

**Figure 1:**
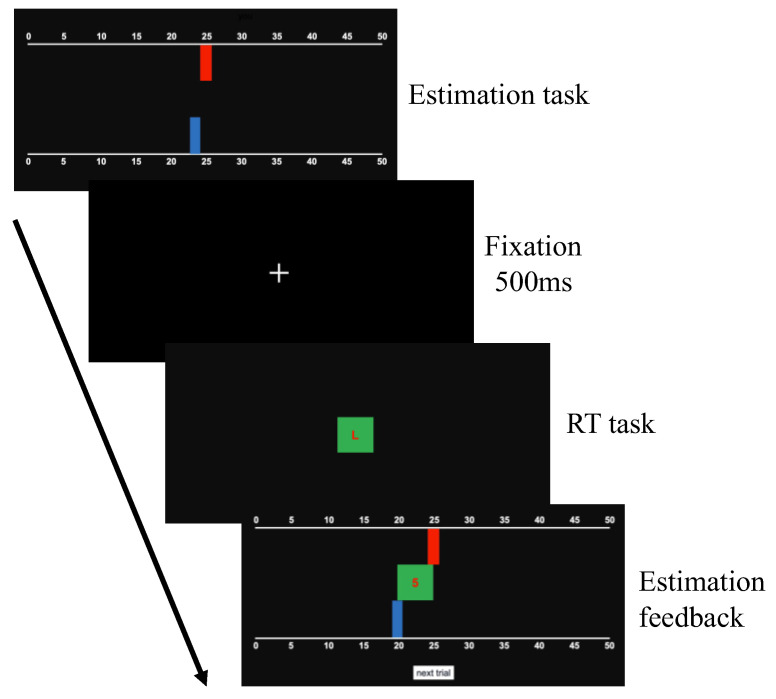
Schematized trial of the estimation task and RT (reaction time) task. First, participants make an estimation (red) by clicking the graduated estimation line. Second, the fixation was shown and lasted for 500 ms. Third, the prediction error (the green rectangle) was shown and one letter (“L” or “R”) was printed on the rectangle. Participants were informed about pressing the left mouse button if the letter is “L” and pressing the right mouse button if the letter is “R” as quickly as possible. After the RT task, the current estimation (the red bar), the newly generated target value (the blue bar), and the prediction error made in the estimation task (the green rectangle) were shown.

A formal block contained one hundred trials, and one trial included two tasks. The first task was an estimation task, where participants were required to predict the target value as closely as possible by clicking the estimation bar (Figure 1 Estimation task). The estimation bar was composed of two graduated lines representing the range of possible numbers (0 to 50) and a blue (0,0,255) bar representing the target value of the latest trial. Participants made estimations by clicking the upper graduated line. After a click, a red (255,0,0) bar, which represented the participants’ estimation in this trial, was printed below the upper graduated line. The prediction error was the absolute difference between the red bar (i.e., the current estimation) and the blue bar (i.e., the new target value). Participants’ task was to minimize prediction errors for all trials in a block.

In the gradually changed block, the distribution of the target value was generated from a Gaussian distribution whose mean changed every ten trials and whose SD was fixed in the block. The mean was 25 (lasted for 20 trials), 30, 35, 30, 25, 20, 15, 20, and 25, respectively. The SD was 1.7. Notably, the 1st trial to the 20th trial, the 31st trial to the 40th trial, the 51st trial to the 60th trial, the 71st trial to the 80th trial, and the 91st trial to the 100th trial were randomly fluctuating trials in the gradually changed block. In other words, the target value fluctuated without a specified trend. The 21st trial to the 30th trial and the 81st to the 90th trial were gradually increased trials, the target value showed a fluctuating upward trend. The 41st trial to the 50th trial, the 61st trial to the 70th trial were gradually decreased trials, the target value was downward fluctuated (shown in Table 1).

In the abruptly changed block, the target value was generated from a Gaussian distribution whose mean changed every twenty trials while holding the SD constant in the block. The mean was 25 (lasted for 30 trials), 35, 25, 15, and 25 (lasted for ten trials), respectively. The SD was 1.7. The first trial of the new distribution was an abruptly changed trial. Thus, the abruptly changed trials in the abruptly changed block were the 30th trial, the 50th trial, the 70th trial, and the 90th trial (shown in Table 1). The rest of the trials were randomly fluctuating trials. In the experiment, we referred to Gino et al. [13] research and set the target value as a sequence of pseudo-random numbers to avoid a potential problem of change mismatch. For example, in the gradually changed block, the latest target value was 22, which was selected from N (25,1.7), and the new target value was 32, which was selected from N (30,1.7). However, the gap between the two values was 10, which was an abrupt change for participants. The advantage of this design is that the target value would not suddenly change when sequential target values were covered by overlapping distributions.

**Figure 2:**
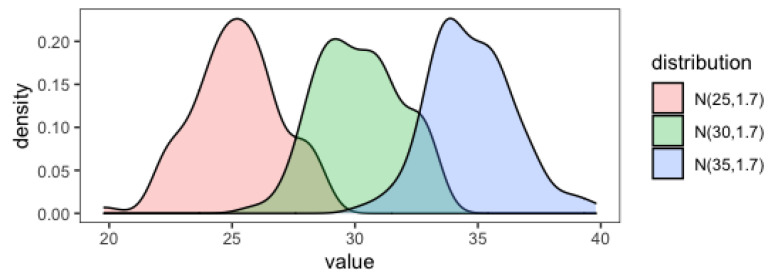
A density plot for the previous distribution and the new distribution. Take the 30th trial as an example. In the gradually changed block, the previous distribution N (30,1.7) (the green density plot) and the new distribution N (35,1.7) (the blue density plot) were highly overlapped. Thus, the change in the target value was relatively smooth. In the abruptly changed block, the previous distribution N (25,1.7) (the pink density plot) and the new distribution N (35,1.7) (the blue density plot) were slightly overlapped; thus, the change of the target value was abrupt and substantial.

The main difference between the gradually changed block and the abruptly changed block was the switching speed of distributions. Take the 10th trial to the 30th trial as an example; in the gradually changed block and the abruptly changed block, the mean of the distribution both began from 25 to 35. In the gradually changed block, as a new distribution N (30,1.7) was inserted, the change in mean was relatively smooth (Figure 2). Hence, the target value was gradually changed. However, in the abruptly changed block, the mean jumped directly from 25 (in the 30th trial) to 35 (in the 31st trial) in a trial. In other words, the blue bar was at 25 in the estimation task and jumped to 35 in the estimation feedback. Therefore, the change in target value abruptly occurred. We predicted that participants would be surprised when they encountered this abrupt change.

The second task was an RT task which aimed to prove that participants were surprised by the significant prediction error in abruptly changed trials. Previous research indicated that RTs would be elevated when participants were surprised [16,25,27,40,41,42]. Thus, significantly increased RTs could be used as evidence to prove that participants were surprised in abruptly changed trials. In the RT task, participants were informed about pressing the corresponding mouse button based on the letter printed on the green (0,255,0) rectangle as quickly as possible. Before the RT task, a fixation display required participants to focus on the center of the screen, which lasted for 500 ms. After the fixation display, a green rectangle was printed on the center of the screen. We informed participants that the width of the green rectangle was consistent with the latest prediction error. The larger the prediction error, the wider the green rectangle. In the RT task, participants pressed the corresponding mouse button based on the letter printed on the center of the green rectangle as quickly as possible. If the letter was “L’’, participants needed to press the left mouse button, and if the letter was “R”, participants were required to press the right mouse button. The probability of the two letters was coincidental. For participants, the main goal of the RT task was to judge the letter in the green rectangle. Therefore, the task-relevant stimulus in this task was the letter, and the width of the green rectangle was the task-irrelevant stimulus. Previous research had observed that participants would be surprised when the task-irrelevant stimulus unexpectedly changed [43,44]. The surprising change attracted participants’ attention from the task-relevant stimulus to the task-irrelevant one, which extended participants’ time to complete the prime task [44]. Therefore, we predicted that if participants were surprised by a sudden increase in the width of the green rectangle, they were likely to shift their attention from the letter in the center of the screen to the green rectangle, which would prolong their reaction time in the RT task. Notably, the RT task in the gradually changed block and the abruptly changed block were identical.

After the RT task, the estimation feedback was displayed. The feedback consisted of a red bar that reflected the current estimation, a blue bar that reflected the new target value, and a green rectangle that reflected the prediction error. Participants were informed to click the “Next trial” button to start a new trial. In a new trial, the target value of the last trial would appear again and be replaced by a new target value in the estimation feedback.

### 4.4. Model and Measured Variables

#### 4.4.1. Model and Measured Variables in the Estimation Task

In the RL model (Equations (1)–(3)), learning is typically driven by the absolute prediction error [35,45]. The degree of learning is quantified by the learning rate [36,37]. A significant learning rate indicates that people put more weight on the new information, i.e., be more susceptible to the new information [36,46]. However, a tiny learning rate suggests that the impact of the new information is less significant [36,47]. Therefore, we used the learning rate to quantify the effect of adjustments in target value on participants’ estimation. If the learning rate increased, it indicated that participants were more affected by the new change. If the learning rate decreased, it suggested that participants were more influenced by the previous belief.

Predicition error_t_ = Target_t_ − Estimate_t_ (1)

Update_t+1_ = Estimate_t+1_ − Estimate_t_ (2)

Update_t+1_ = learning rate ∗ Prediction error_t_ (3)

There are two important variables in the RL model which were measured in the estimation task. The first variable was the prediction error which was the difference between the target value and the participants’ reported estimation (Equation (1)) [48,49,50,51].

The second variable was the updated value which was the difference between the latest estimation and the new estimation (Equation (2)).

In addition, we calculated participants learning rates by using the prediction error and the updated value (Equation (3)). By comparing the learning rate in gradually changed trials and abruptly changed trials, we could demonstrate participants’ different sensitivity toward different changes.

#### 4.4.2. Measure Variables in the RT Task

In the RT task, we recorded participants’ reaction time from seeing a letter on the center of the screen to clicking the corresponding mouse button. The RTs were to be analyzed to confirm that participants were surprised by large prediction errors.

### 4.5. Apparatus

All experiments were conducted using the computer software package Psychtoolbox 3 (PTB-3) in MATLAB 2017b [52,53]. Stimuli were presented on a 19.5-in LED monitor with a solution of 1600 ∗ 900 pixels and a refresh rate of 75 Hz, which was attached to an (Intel Xeon) personal computer (Lenovo, Beijing, China). Responses were recorded using a mouse.

## 5. Results

**Figure 3:**
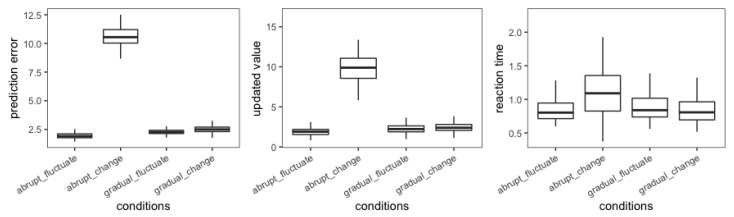
Box plot of distributions of means under different conditions.

Before testing the hypotheses, we briefly summarized the variable we recorded in the experiment. It included the variables measured in the estimation task (i.e., the prediction error and the updated value) and the RT task (i.e., the RTs). First, the average prediction error in gradually changed trials was 2.558, while it was 10.521 in abruptly changed trials. It showed that participants were more likely to make large prediction errors when the target value was abruptly changed (Table 2 and Figure 3). Second, the average updated value in gradually changed trials and abruptly changed trials was 2.400 and 9.827, respectively. It demonstrated that participants tended to significantly update their estimation when the target value abruptly changed (Table 2 and Figure 3). Finally, the average RTs in gradually changed trials was 0.85 which was obviously smaller than the RTs in abruptly changed trials (1.105). It suggested that participants were more surprised by the prediction error when the target value suddenly adjusted (Table 2 and Figure 3).

Hypothesis 1: The learning rate is relatively low when participants face a sequence of gradual changes. However, the learning rate is high when participants encounter an abrupt change.

We performed a mixed-linear regression to estimate participants’ learning rates under different conditions. According to the RL model, the updated value is the difference between the previous and new estimates. The prediction error is the discrepancy between the new estimation and the new target value, and the coefficient of the prediction error is the learning rate. To contrast the learning rate under different conditions, we set the updated value as the dependent variable; the prediction error and the change mode (abruptly change/randomly fluctuate in the abrupt block/gradually change/randomly fluctuate in the gradual block (baseline)) as independent variables The model is:

update = β_0_ + β_1_ ∗ prediction error + β_2_ ∗ change mode + β_3_ ∗ prediction error ∗ change mode

The result demonstrated that participants’ average learning rate was 0.528 (*β* = 0.528, *p* < 0.001 ***) when the target value randomly fluctuated in the gradually changed block. When the target value gradually changed, the average learning rate increased by 0.005 (*p* = 0.748). However, this increase was not significant. Therefore, the average learning rate in gradually changed trials was 0.528 as well. When the target value randomly fluctuated in the abruptly changed block, the learning rate increased by 0.067 (*β* = 0.067, *p* < 0.001 ***). Thus, the learning rate was 0.595. When the change abruptly occurred, the average learning rate soared by 0.165 (*β* = 0.165, *p* < 0.001 ***) (See more details in Table 3). In other words, the learning rate was 0.693 in abruptly changed trials. This result confirmed that participants’ learning rate increased when they encountered an abrupt change, and the learning rate was relatively low when changes were gradually introduced.

Hypothesis 2: Participants’ learning rates are associated with their prediction errors. Under the abruptly changed condition, the learning rates show a significant value jump due to the large prediction error produced by the participants. In addition, the large prediction error more likely occurs in abruptly changed trials.

We confirmed that participants could not detect the change when the prediction error was tiny. Thus, the learning rate was low and participants would realize the change occurred when the prediction error was large enough to trigger surprise, which resulted in a soaring learning rate. Based on the cognitive-evolutionary model of surprise, we proposed that the learning rate increased when the prediction error exceeded the threshold. In other words, prediction error and updated value were nonlinearly related. Thus, we could test two hypotheses in the following section: first, we confirmed that the relation between prediction error and update value was not linear; second, we used segmented mixed models to estimate the location of the average breakpoint for all participants. We expected the learning rate to increase when the prediction error exceeded the breakpoint and decrease when the prediction error was below the breakpoint.

To verify that the relationship between the updated value and prediction error was not linear, we fitted a quadratic regression model, which is commonly used for detecting nonlinear effects [54]. The model is

update = β_0_ + β_1_ ∗ prediction error + β_2_ ∗ prediction error^2^

As the result shown in Table 4. The coefficient of the quadratic term was 0.032 (*p* < 0.001). It indicated that the updated value and prediction error had a nonlinear relationship.

We expected that the learning rate in large prediction error trials and small prediction error trials would be different. The learning rate increased when the prediction error was above the breakpoint. To locate the breakpoint and estimate the learning rate above and below the threshold, we conducted a segmented mixed model with random changepoints [55] in R. The model is

update = β_0_ + β_1_ ∗ prediction error + δ ∗ (prediction error − ψ) + ε

The result was shown in Table 5. It indicated that the breakpoint is 5.337. When the prediction error was above this value, the learning rate increased by 0.536.

Moreover, we conducted a logistic regression to investigate the odds that prediction errors were above the threshold under different conditions (Table 6). The result indicated that participants tended to make significant prediction errors when the target value abruptly changed (*β* = 7.999, *p* < 0.001 ***).

**Figure 4:**
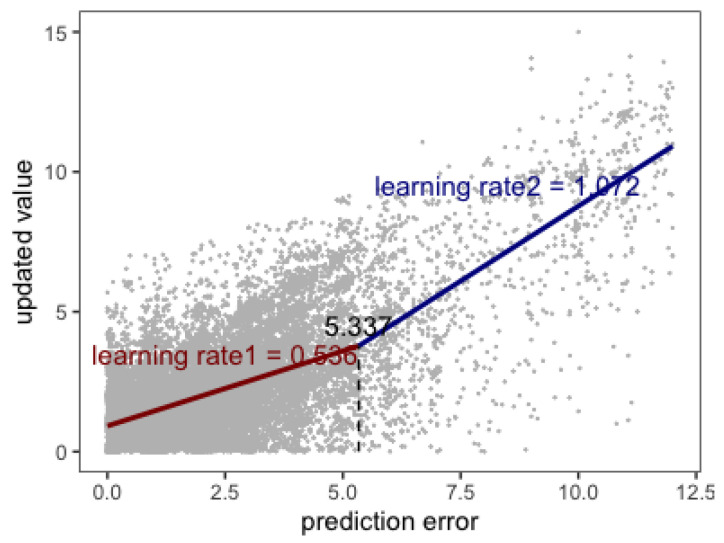
Segmented mixed regression.

We proved that the learning rate was not a constant in this experiment. The learning rate was relatively low when the prediction error was small and increased when the prediction error exceeded the threshold (see Table 5 and Figure 4). The target value slightly changed under gradually changing conditions (including randomly fluctuated trials in the abruptly changed block and the gradually changed block). Therefore, participants could make relatively accurate predictions after a period of learning. As a result, the probability of making a significant prediction error was very low (see Table 6), which led to the learning rate being small in gradually changed trials. However, when the target value abruptly changed, it was very likely that participants made a significant prediction error (see Table 6) which induced the surprise emotion and led to an increase in learning rate (see Table 5 and Figure 4). This could explain why participants were more sensitive to abrupt changes.

Hypothesis 3: Participants are surprised by the significant prediction error in abruptly changed trials because RTs are elevated in these trials.

Before RT analysis, RT longer than 3 seconds was excluded. We conducted a mixed linear model to confirm that RTs were elevated in abruptly changed trials. A significantly elevated RT proved that participants were surprised by abrupt changes. RT was used as the dependent variable, change mode (abruptly change/randomly fluctuate in the abrupt block/gradually change/randomly fluctuate in the gradual block (baseline)) was used as the independent variable, and participants were used as the random variable. The model is as follows:

RTs = β_0_ + β_1_ ∗ change mode

The result was summarized in Table 7. It showed that abrupt changes significantly prolonged participants’ RTs (*β* = 0.222, *p* < 0.001 ***), which indicated that participants were surprised by abrupt changes. Hypothesis 3 was supported.

## 6. Discussion

Previous research indicated that participants were imperceptive to gradual changes while perceptive to sudden and substantial changes [13,14,15]. This phenomenon was called the frog-in-the-pan phenomenon (FIP). Based on the cognitive-evolutionary model of surprise, we proposed that the gap between participants’ expectation and their observations was large enough to trigger surprise emotion when the target value abruptly changed. Therefore, participants were more sensitive to abrupt changes. To test our prediction, we first compared participants’ learning rates when the target value progressively and suddenly adjusted. The result suggested that participants’ learning rate soared by 0.165 when the target value suddenly changed and proved that the FIP phenomenon existed in our research. Second, by conducting mixed segmented regression and logistic regression, we confirmed that the learning rate increased by 0.536 when the prediction error was above 5.337 and large prediction errors tended to occur in abruptly changed trials (exp(β) = 2976.766). Hence, learning rates were more likely to increase when the target value suddenly adjusted. We conjectured that surprise led to an increase in learning rates when the prediction error was significant. The cognitive-evolutionary model of surprise could explain why participants are more likely to update their beliefs when the prediction error is large [23]. This theory states that participants would be aware that their expectations are significantly different from their observations and would update their beliefs when they are surprised. For the previous belief to be invalid, people need to rely more on the new information. As a result, participants’ learning rate is increased when the prediction error is large. In a word, the theory suggests that surprise is the key to changing participants’ sensitivity to the change in target value [23].

In addition, previous research showed that surprise accelerated the learning rate and led participants to be more attentive to adjustments in target value. First, when the target value abruptly changed, the prediction error immediately soared, which elicited participants’ surprise emotion and attracted more cognitive processing to it [56]. Thus, a higher “weight” was put on the new information when participants made a recent estimation [57]. Second, people would exhibit surprise-driven learning when they were surprised. They were more likely to believe that the context had been changed, and information obtained before the surprise was no longer helpful in making predictions [17,33,58]. Therefore, people would discard their prior beliefs and emphasize newly arriving information, which led to participants being more sensitive to abrupt changes. We also find some neuro-image evidence to support these abovementioned processes in the neuroscience field. Previous research indicated that when people were surprised by the discrepancy between their beliefs and newly acquired information, the right anterior insula would activate the central executive network and deactivate the default mode network to initiate attentional control, which resulted in more cognitive resources being allocated to process surprising events [58]. In addition, previous research indicated that signal change in the dorsal anterior cingulate cortex (dACC) could predict the average learning rate across individuals [36]. dACC would be significantly activated when it detected that the belief was no longer supported by evidence from the environment [58,59,60]. As a result, people would have a higher learning rate and give more weight to the new information [36]. These factors both drove overreaction when the target value abruptly changed.

In this research, we observed that surprise emotion resulted in belief updating. However, it did not indicate that participants were bound to update their estimations when surprised. First, previous studies have found that the learning rate varied from person to person. Some participants did not change their estimation even if the new outcome was far different from their expectations [17], which was also discovered in our research. Second, the surprising event that participants encountered in this study was informative. In other words, the surprise was a signal to inform participants to update their estimation [61,62]. However, in the first abruptly changed trial, participants could not confirm whether the surprise was informative or not. Hence a minority of them did not update their estimation and stuck to their previous strategy. As they came to realize that the abrupt change was informative, they tended to update their estimation. Some existing studies in cognitive psychology have confirmed that people tend to stick to their previous estimation if a surprise is uninformative. Under this circumstance, people felt surprised and prolonged their RTs, but did not revise their beliefs. Besides, fMRI images showed that the learning-related brain region did not strongly activate [61,62]. It indicated that neither participants changed their estimations nor were sensitive to the surprise when it was uninformative.

Compared to previous research, we first provided a new explanation for the FIP phenomenon from the perspective of surprise emotion. However, our research shared many similarities with existing research on the FIP phenomenon. As we mentioned, Offerman and his colleagues set the subsidy to gradually change and quickly change in their public goods game to investigate the FIP phenomenon [15]. When the subsidy quickly increased to 0.75 in a short period, participants’ contributions jumped by 2.86, while the contributions increased by 0.73 when the subsidy progressively increased. It revealed that participants were indeed more perceptive to sudden and substantial changes. In other words, the FIP phenomenon exists in the public goods game. Notably, Offerman et al. provided an explanation for the FIP phenomenon based on the “thinking cost”. They believed that participants tended to adjust their strategy when they detected a change in the environment. However, they did not elaborate on how people notice the situation has changed. In this study, we proved that the large prediction error triggered surprise emotion, people thus came to realize that the previous belief is invalid and updated their belief. Surprise emotion was the key to making people aware that the environment has changed significantly.

In this study, we observed participants’ reaction time was shorter when the target value progressively adjusted, and the reaction time was longer when the target value abruptly adjusted (mean 0.85 vs. 1.105). This result indicated that participants were surprised when the target value suddenly changed. The cognitive-evolutionary model of surprise proposed that surprising events attract attention and cognitive resources [23]. If participants are surprised, they will allocate more attention and cognitive resources to the surprising event [27]. When the prediction error unexpectedly increased, the attention and the cognitive resources which had been assigned to the letter (“L” or “R”) were diverted to processing the surprising prediction error. As a result, RTs were extended in abruptly changed trials.

Yang also measured the reaction time when subjects received an abrupt change or a series of gradual changes [19]. She found that participants’ reaction time was longer when participants acquired a sequence of small changes while the reaction time was shorter when they received a dramatic change (exp1: t(34.975) = 2.474, *p* = 0.018; exp2: t(40.128) = 2.493, *p* = 0.017). At a first glance, the conclusion of this research seems contradictory to Yang. However, in Yang’s research, the reaction time reflected how long participants took to make a decision after receiving the information. The results showed that it was more difficult for subjects to make decisions after a sequence of gradual changes because they did not know whether the context had changed. And they could more easily make decisions after a dramatic change as they noticed the change. In this study, the reaction time reflected how long participants responded to the prediction error. We observed that reaction time was prolonged when participants encountered a large prediction error. The large prediction error triggered surprise emotion which implied the situation had changed, and the reaction time was thus prolonged. In a word, even though reaction times in the two studies were obviously different, they both proved that participants detected the change in the context. Therefore, the conclusions of the two studies were not contradictory.

In this study, we proposed that surprise emotion could explain the FIP in the financial market and in daily life. In previous research, Choi and Hui proved that surprise could explain the overreaction of investors in the stock market and they verified their hypothesis by analyzing the data in the betting market [57]. They stated that a surprising event was hard to comprehend which led people to put more weight on the unintended event. This opinion was consistent with the explanatory difficulty hypotheses [63,64]. In future studies, we will research how to use the explanatory difficulty hypothesis to explain the FIP as well.

In this research, we provide a new explanation for the frog-in-the-pan phenomenon. We demonstrated that the difference in sensitivity towards gradual and abrupt changes is due to the elicitation of surprise emotion rather than a lack of attention. Notably, our explanation did not suggest that surprise emotion was the only factor that resulted in different sensitivity toward different adjustments. Our explanation is more suitable for circumstances that require people to form expectations. For example, we purchase milk every day and form an expectation of its price through repeated purchases. When the milk price changes gradually, people tend to be more insensitive to the new price and believe that the milk price is steady. However, when the price changes abruptly, people realize that the price has substantially changed. Nevertheless, overestimation and underestimation of a price could have a different effect on the following decision, and we will investigate the difference in future work.

## Figures and Tables

**Table 1:** The distribution of target values in two formal blocks.

Gradually Changed Block (Including Gradually Increase/Decrease Trials)			Abruptly Changed Block (Including Abruptly Increase/Decrease Trials)		
Trials	Distribution	Trend	Trials	Distribution	Trend
1–20	N (25,1.7)	randomly fluctuate	1–29	N (25,1.7)	randomly fluctuate
21–30	N (30,1.7)	gradually increase	30	N (35,1.7)	abruptly increase
31–40	N (35,1.7)	randomly fluctuate	31–49		randomly fluctuate
41–50	N (30,1.7)	gradually decrease	50	N (25,1.7)	abruptly decrease
51–60	N (25,1.7)	randomly fluctuate	51–69		randomly fluctuate
61–70	N (20,1.7)	gradually decrease	70	N (15,1.7)	abruptly decrease
71–80	N (15,1.7)	randomly fluctuate	71–89		randomly fluctuate
81–90	N (20,1.7)	gradually increase	90	N (25,1.7)	abruptly increase
91–100	N (25,1.7)	randomly fluctuate	91–100		randomly fluctuate

**Table 2:** Descriptive statistics for variables in the experiment.

	Prediction Error (sd)	Updated Value (sd)	RTs (sd)
randomly fluctuate in the gradual block	2.316 (1.636)	2.238 (1.709)	0.883 (0.403)
gradually change	2.558 (1.825)	2.400 (1.772)	0.85 (0.384)
randomly fluctuate in the abrupt block	1.985 (1.570)	1.935 (1.554)	0.855 (0.355)
abruptly change	10.521 (1.933)	9.827 (2.419)	1.105 (0.471)

**Table 3:** The learning rate in the gradually changed block and the abruptly changed block: Results of mixed-linear model.

	*β*	SE	df	t Value	Pr (>|t|)
(Intercept)	1.017	0.047	250.1	21.742	<0.001 ***
prediction error	0.528	0.01	21,113.526	51.731	<0.001 ***
gradually change	0.022	0.045	21,089.861	0.48	0.631
randomly fluctuate in the abrupt block	−0.261	0.036	21,096.218	−7.311	<0.001 ***
abruptly change	1.531	0.361	21,090.713	4.246	<0.001 ***
prediction error × gradually change	0.005	0.015	21,093.512	0.322	0.748
prediction error × randomly fluctuates in the abrupt block	0.067	0.013	21,102.968	5.06	<0.001 ***
prediction error × abruptly change	0.165	0.035	21,094.483	4.71	<0.001 ***

Note: *** *p* < 0.001.

**Table 4:** The result of quadratic regression model.

	*β*	SE	df	t Value	Pr (>|t|)
(Intercept)	1.009	0.041	152.078	24.63	<0.001 ***
prediction error	0.405	0.011	21,112.450	38.38	<0.001 ***
prediction error^2^	0.032	0.001	21,102.695	29.39	<0.001 ***

Note: *** *p* < 0.001.

**Table 5:** The result of the segmented mixed model.

*β*_0_	*β*_1_	δ	Breakpoint
0.916	0.536	0.536	5.337

**Table 6:** The result of logistic regression.

	*β*	SE	z Value	Pr (>|z|)	Exp (*β*)
(Intercept)	−3.459	0.104	−33.134	<0.001 ***	0.031
gradually change	0.575	0.088	6.524	<0.001 ***	1.777
randomly fluctuate in the abrupt block	−0.361	0.087	−4.158	<0.001 ***	0.697
abruptly change	7.999	0.434	18.417	<0.001 ***	2976.766

Note: *** *p* < 0.001.

**Table 7:** The result of the mixed linear model of RT.

	*β*	SE	df	t Value	Pr ( > |t|)
(Intercept)	0.885	0.018	116.939	49.601	<0.001 ***
gradually change	−0.034	0.007	20,900.043	−5.182	<0.001 ***
randomly fluctuate in the abrupt block	−0.028	0.005	20,900.093	−5.261	<0.001 ***
abruptly change	0.221	0.017	20,900.295	13.041	<0.001 ***

Note: *** *p* < 0.001.

## Data Availability

The data that support the finding of this study are available from the corresponding author, upon request.

## References

[1] Fama E.F. (1970). Efficient capital markets: A review of theory and empirical work. J. Financ..

[2] Konstantinidis A., Katarachia A., Borovas G., Voutsa M.E. (2012). From efficient market hypothesis to behavioural finance: Can behavioural finance be the new dominant model for investing. Sci. Bull. Econ. Sci..

[3] Kumar S., Goyal N. (2016). Evidence on rationality and behavioural biases in investment decision making. Qual. Res. Financ. Mark..

[4] Sadi R., Asl H.G., Rostami M.R., Gholipour A., Gholipour F. (2011). Behavioral finance: The explanation of investors’ per-sonality and perceptual biases effects on financial decisions. Int. J. Econ. Financ..

[5] Muhammad N.M.N. (2009). Study on behavioral finance: Is the individual investors rational. Adv. Manag..

[6] Bloomfield R., O’Hara M., Saar G. (2009). How Noise Trading Affects Markets: An Experimental Analysis. Rev. Financ. Stud..

[7] Poteshman A.M., Serbin V. (2003). Clearly Irrational Financial Market Behavior: Evidence from the Early Exercise of Exchange Traded Stock Options. J. Financ..

[8] Karlsson A., Nordén L. (2007). Home sweet home: Home bias and international diversification among individual investors. J. Bank. Financ..

[9] Uchida H., Nakagawa R. (2007). Herd behavior in the Japanese loan market: Evidence from bank panel data. J. Financ. Intermediation.

[10] Chau F., Deesomsak R., Koutmos D. (2016). Does investor sentiment really matter?. Int. Rev. Financ. Anal..

[11] Kumari J., Mahakud J. (2015). Does investor sentiment predict the asset volatility? Evidence from emerging stock market India. J. Behav. Exp. Financ..

[12] Liang L. (2003). Post-Earnings Announcement Drift and Market Participants’ Information Processing Biases. Rev. Account. Stud..

[13] Gino F., Bazerman M.H. (2009). When misconduct goes unnoticed: The acceptability of gradual erosion in others’ unethical behavior. J. Exp. Soc. Psychol..

[14] Da Z., Gurun U.G., Warachka M. (2014). Frog in the Pan: Continuous Information and Momentum. Rev. Financ. Stud..

[15] Offerman T., van der Veen A. (2015). How to subsidize contributions to public goods: Does the frog jump out of the boiling water?. Eur. Econ. Rev..

[16] Meyer W.-U., Reisenzein R., Schützwohl A. (1997). Toward a Process Analysis of Emotions: The Case of Surprise. Motiv. Emot..

[17] Nassar M., Wilson R.C., Heasly B., Gold J.I. (2010). An Approximately Bayesian Delta-Rule Model Explains the Dynamics of Belief Updating in a Changing Environment. J. Neurosci..

[18] Huang S., Lee C.M., Song Y., Xiang H. (2022). A frog in every pan: Information discreteness and the lead-lag returns puzzle. J. Financ. Econ..

[19] Yang W.T. (2019). The Influence of Information Presenting on Individual’s Investment Decision-Making: Emotional and Neuroscience Approach.

[20] Piccoli P., Chaudhury M., Souza A., da Silva W.V. (2017). Stock overreaction to extreme market events. North Am. J. Econ. Financ..

[21] Griffin D., Tversky A. (1992). The weighing of evidence and the determinants of confidence. Cogn. Psychol..

[22] Ekman P., Friesen W.V. (1971). Constants across cultures in the face and emotion. J. Pers. Soc. Psychol..

[23] Reisenzein R., Horstmann G., Schützwohl A. (2017). The Cognitive-Evolutionary Model of Surprise: A Review of the Evidence. Top. Cogn. Sci..

[24] Schützwohl A. (2018). Approach and Avoidance During Routine Behavior and During Surprise in a Non-evaluative Task: Surprise Matters and So Does the Valence of the Surprising Event. Front. Psychol..

[25] Schützwohl A. (1998). Surprise and schema strength. J. Exp. Psychol. Learn. Mem. Cogn..

[26] Reisenzein R. (2000). Exploring the Strength of Association between the Components of Emotion Syndromes: The Case of Surprise. Cogn. Emot..

[27] Niepel M., Rudolph U., Schützwohl A., Meyer W.-U. (1994). Temporal characteristics of the surprise reaction induced by schema-discrepant visual and auditory events. Cogn. Emot..

[28] Smedslund J. (1990). A critique of Tversky and Kahneman’s distinction between fallacy and misunderstanding. Scand. J. Psychol..

[29] Ekman P. (2003). Emotions Revealed. Understanding Faces and Feelings.

[30] Teigen K.H., Keren G. (2003). Surprises: Low probabilities or high contrasts?. Cognition.

[31] Plutchik R. (1980). Emotion: A Psychoevolutionary Synthesis.

[32] Meyer W.U., Niepel M., Rudolph U., Schützwohl A. (1991). An experimental analysis of surprise. Cogn. Emot..

[33] McGuire J.T., Nassar M.R., Gold J.I., Kable J.W. (2014). Functionally Dissociable Influences on Learning Rate in a Dynamic Environment. Neuron.

[34] Ofir C., Mazursky D. (1997). Does a Surprising Outcome Reinforce or Reverse the Hindsight Bias?. Organ. Behav. Hum. Decis. Process..

[35] Sutton R.S., Barto A.G. (2017). Reinforcement Learning: An Introduction.

[36] Behrens T.E.J., Woolrich M.W., E Walton M., Rushworth M. (2007). Learning the value of information in an uncertain world. Nat. Neurosci..

[37] Bossaerts P. (2018). Formalizing the Function of Anterior Insula in Rapid Adaptation. Front. Integr. Neurosci..

[38] Reisenzein R., Bördgen S., Holtbernd T., Matz D. (2006). Evidence for strong dissociation between emotion and facial displays: The case of surprise. J. Pers. Soc. Psychol..

[39] Horstmann G. (2005). Attentional Capture by an Unannounced Color Singleton Depends on Expectation Discrepancy. J. Exp. Psychol. Hum. Percept. Perform..

[40] Kvålseth T.O. (1987). Stimulus probability, surprise, and reaction time. Proceedings of the Human Factors Society Annual Meeting.

[41] Mars R.B., Debener S., Gladwin T.E., Harrison L.M., Haggard P., Rothwell J.C., Bestmann S. (2008). Trial-by-trial fluctuations in the event-related electroencephalogram reflect dynamic changes in the degree of surprise. J. Neurosci..

[42] Schützwohl A., Borgstedt K. (2005). The processing of affectively valenced stimuli: The role of surprise. Cogn. Emot..

[43] Horstmann G., Becker S.I. (2011). Evidence for goal-independent attentional capture from validity effects with unexpected novel color cues—A response to Burnham (2007). Psychon. Bull. Rev..

[44] Horstmann G., Herwig A. (2014). Surprise attracts the eyes and binds the gaze. Psychon. Bull. Rev..

[45] Rescorla R.A. (1972). A theory of Pavlovian conditioning: Variations in the effectiveness of reinforcement and nonreinforcement. Curr. Res. Theory.

[46] Bai Y., Katahira K., Ohira H. (2014). Dual learning processes underlying human decision-making in reversal learning tasks: Functional significance and evidence from the model fit to human behavior. Front. Psychol..

[47] Wu X., Wang T., Liu C., Wu T., Jiang J., Zhou D., Zhou J. (2017). Functions of Learning Rate in Adaptive Reward Learning. Front. Hum. Neurosci..

[48] Bayer H.M., Glimcher P.W. (2005). Midbrain Dopamine Neurons Encode a Quantitative Reward Prediction Error Signal. Neuron.

[49] Glimcher P.W. (2011). Understanding dopamine and reinforcement learning: The dopamine reward prediction error hypothesis. Proc. Natl. Acad. Sci. USA.

[50] Schultz W. (2016). Dopamine reward prediction error coding. Dialog- Clin. Neurosci..

[51] Eshel N., Tian J., Bukwich M., Uchida N. (2016). Dopamine neurons share common response function for reward prediction error. Nat. Neurosci..

[52] Brainard D.H., Vision S. (1997). The psychophysics toolbox. Spat. Vis..

[53] Kleiner M., Brainard D., Pelli D. (2007). What’s New in Psychtoolbox-3?. https://pure.mpg.de/rest/items/item_1790332/component/file_3136265/content.

[54] Ren D., Stavrova O., Loh W.W. (2022). Nonlinear effect of social interaction quantity on psychological well-being: Diminishing returns or inverted U?. J. Pers. Soc. Psychol..

[55] Muggeo M.R. (2016). Segmented Mixed Models with Random Changepoints in R. https://www.researchgate.net/publication/292629179_Segmented_mixed_mo-dels_with_random_changepoints_in_R.

[56] Topolinski S., Strack F. (2015). Corrugator activity confirms immediate negative affect in surprise. Front. Psychol..

[57] Choi D., Hui S.K. (2014). The role of surprise: Understanding overreaction and underreaction to unanticipated events using in-play soccer betting market. J. Econ. Behav. Organ..

[58] Stöttinger E., Aichhorn M., Anderson B., Danckert J. (2018). The neural systems for perceptual updating. Neuropsychologia.

[59] Domenech P., Koechlin E. (2015). Executive control and decision-making in the prefrontal cortex. Curr. Opin. Behav. Sci..

[60] McGuire J.T., Kable J.W. (2015). Medial prefrontal cortical activity reflects dynamic re-evaluation during voluntary persistence. Nat. Neurosci..

[61] O’Reilly J.X., Schüffelgen U., Cuell S.F., Behrens T.E.J., Mars R.B., Rushworth M.F.S. (2013). Dissociable effects of surprise and model update in parietal and anterior cingulate cortex. Proc. Natl. Acad. Sci. USA.

[62] Nour M.M., Dahoun T., Schwartenbeck P., Adams R.A., FitzGerald T.H.B., Coello C., Wall M.B., Dolan R.J., Howes O.D. (2018). Dopaminergic basis for signaling belief updates, but not surprise, and the link to paranoia. Proc. Natl. Acad. Sci. USA.

[63] Foster M.I., Keane M.T. (2015). Why some surprises are more surprising than others: Surprise as a metacognitive sense of explanatory difficulty. Cogn. Psychol..

[64] Maguire R., Maguire P., Keane M.T. (2011). Making sense of surprise: An investigation of the factors influencing surprise judgments. J. Exp. Psychol. Learn. Mem. Cogn..

